# Individual Variables Involved in Perceived Pressure for Adolescent Drinking

**DOI:** 10.3390/ijerph17062012

**Published:** 2020-03-18

**Authors:** María del Carmen Pérez-Fuentes, María del Mar Molero Jurado, José Jesús Gázquez Linares, África Martos Martínez, Isabel Mercader Rubio, Mahia Saracostti

**Affiliations:** 1 Department of Psychology, University of Almería, 04120 Almería, Spain; mpf421@ual.es (M.d.C.P.-F.); amm521@ual.es (Á.M.M.); imercade@ual.es (I.M.R.); 2 Department of Psychology, Universidad Autónoma de Chile, 4780000 Santiago, Chile; 3 Escuela de Trabajo Social, Facultad de Ciencias Sociales, Universidad de Valparaíso, 2653 Valparaíso, Chile; mahia.saracostti@ufrontera.cl

**Keywords:** perceived pressure, alcohol use, self-esteem, anxiety sensitivity, adolescence, impulsivity, expectations

## Abstract

Adolescence is a stage when individuals are especially vulnerable to the influence of their peer group, which could lead to the development of problematic behavior, such as drinking alcohol, due to perceived pressure. The objective of this study was to analyze the role of self-esteem, impulsivity, anxiety sensitivity and expectations for use under perceived pressure to drink alcohol among young people. Methods: The sample was made up of 1287 high school students aged 14 to 18, with a mean age of 15.11. The Bayes factor and mediation models were estimated to evaluate the data. Results: The results showed the existence of a positive relationship of impulsivity, anxiety sensitivity and expectations for use with perceived pressure. However, this relationship was negative with self-esteem and perception of pressure to drink alcohol. Furthermore, the model results showed that self-esteem mediates the relationship between physical, cognitive and social anxiety sensitivity and positive expectations with perceived pressure to drink alcohol in adolescence. Conclusions: Given the strong need for affiliation during youth, it is hard to control grouping and peer influence on drinking behavior. However, knowledge of the role of individual variables, such as those described here, in perceived pressure could improve the prevention and intervention of such behaviors.

## 1. Introduction

### 1.1. Adolescent Alcohol Use

Alcohol is the psychoactive substance most widely used by high school students [1]. Its use is especially problematic because in adolescence the brain is particularly vulnerable to the neurotoxic effects of alcohol [2,3]. Drinking during puberty has been associated with brain impairments in adulthood, which leads to a higher risk of developing alcohol use and anxiety disorders following youth [4,5] Other dangers to health associated with alcohol use are at-risk sexual behaviors, polysubstance use or sleep impairment [6,7,8,9]. Along with the implications for health, the use of alcohol by adolescents is related to decreased school participation, verbal learning, attention and memory impairments, moral detachment, the appearance of problematic behaviors and violence [10,11,12,13,14].

For prevention, parental education exerts influence on their children’s drinking behavior [15]. However, the impact of drinking by the peer group may be even greater [16], as young people begin to differentiate themselves from the family and develop ever closer affiliations with their peers [17,18]. Thus, parental influence on the development of problematic behavior, such as violence or substance use, is mediated by affiliation with peers with that behavior [19]. 

### 1.2. Perceived Pressure to Drink Alcohol

Adolescent drinking is marked by beliefs about the social consequences associated with it, which originate in social and environmental factors to the detriment of the individual’s biological characteristics [20]. At this stage, there is a reciprocal relationship of social influence with social selection. Thus, through social influence, the peer group affects drinking, and, through social selection, the drinking behavior has repercussions on those chosen as friends [21]. The most grouping is therefore found when the levels of influence and social selection are highest, which in turn, leads to drinking in groups being either very high or practically non-existent, with few individuals grouped at intermediate levels of alcohol use [22]. For example, as shown in the study by Rossheim et al. [23], adolescents who drink with others are more likely to have episodes of excessive drinking. 

Along this line, with regard to perceived pressure, the influence of classmates, and even more so, of the group of friends, on drinking behavior is related to the feeling of group identity. Young people select groups with similar behavior, which later influence how they drink alcohol [24]. Therefore, how much adolescents drink tends to be associated with how much their peers do [25], (especially best friends [18], and how much influence depends on the individual’s resistance to the influence of classmates [26]. Thus, complacence to peer pressure to drink alcohol is a causal variable in adolescent drinking behavior [15], and even more so considering that young people tend to overestimate the participation of their peers in risk behaviors such as drinking, which then affects their own [27]. On the contrary, insofar as retraction, or quitting, during adolescence, the perceived increase in drinking by the peer group, approval of drinking by peers and expectations for alcohol predict a lower probability of stopping [28,29]. 

### 1.3. Risk Variables for Resisting Use

Along with social factors, cognitive factors, such as impulsivity, emotional intelligence and sensation seeking, have been shown to be as important as perceived pressure from the peer group in predicting legal substance use [30,31,32]. Impulsivity has specifically been postulated as a strong predictor of alcohol use by adolescents [33]. Impulsivity, which refers to the tendency to react quickly, without considering the consequences [34], is an individual development trait, and every individual can be characterized by their impulsive tendencies [35]. High impulsivity has been structurally associated with a reduction in the thickness of cortical structures, such as frontal and orbital areas and middle frontal gyrus [34]. Even though the pattern of cortical thickening decreases rapidly during early adolescence, followed by more gradual thinning to stabilization during adulthood [36], the changes in impulsive youths cannot be associated with changes related to the synaptic pruning typical of this stage of development [37]. High levels of impulsivity have been associated with risk-taking and with starting and increasing substance use in adolescence [30,38,39,40,41,42]. This use shows a stronger association with impulsivity when it is oriented toward sensation seeking (that is, the desire to experience new sensations and forms of entertainment), and positive or negative urgency (in other words, the tendency to act impulsively in an exceptionally good or bad mood, respectively) [43]. However, some authors suggest that the impulsivity associated with mood is only related to adolescent drinking in negative urgency [44]. Concerning the path of continued drinking or polysubstance use, adolescents who begin early show higher impulsivity than young people with experimental, non-continuous use of such substances [45]. In addition, with regard to group pressure and impulsivity in drinking alcohol during adolescence, Baumeister and Vonasch [46], found that the increase in impulsivity levels along with the reduction in the capacity for self-regulation in this stage of development increase vulnerability to yielding to influence in substance use. 

Along with impulsivity, another variable involved in adolescent drinking is expectations. Positive expectations about the results of drinking by adolescents minimize the perception of risk and facilitate the startup and maintenance of drinking habits [47]. For example, in the study by Gibbons et al. [48], after showing videos of the positive consequences (social facilitation) associated with drinking alcohol to a group of adolescents with high impulsivity scores, their disposition to use increased. Thus, expectations about alcohol are important in explaining the drinking behavior of young people [49]. The perception of drinking alcohol as a way of relaxing and eliminating social inhibition, therefore, forms part of the positive expectations associated with alcohol which are most repeated in adolescent discourse [24]. With regard to social stimulation, after drinking alcohol, young people perceive themselves to be more excited, talkative and active. These expectations are not as high among older drinkers [50]. On the contrary, negative expectations are an intrinsic motivation for the adolescent to seek professional help when they perceive a problem of abuse with a substance such as alcohol [51]. Positive expectations of drinking increase over time in minors and are predictors of the beginning of drinking alcohol in adolescence and developing a problematic drinking pattern [12,49]. This is partly due to such expectations creating an attentional bias that promotes perception and memory of desirable consequences associated with drinking [52]. Similarly, having positive expectations for alcohol generates a higher risk of future use among the youngest adolescents, both directly and through its impact on self-efficacy of refusing to drink. Thus, favorable beliefs about drinking weaken adolescent self-efficacy in resisting the use of a substance they think will lead them to gratifying social consequences [53]. 

Anxiety sensitivity is another individual variable that has been shown to be related to adolescent use of alcohol. This variable, which affects one’s wellbeing and happiness [54], has been defined as the fear of and agitation caused by anxiety-related sensations [55], and is made up of three factors, which refer to concern for physiological alteration, deviant thinking and social evaluation situations, called sensitivity to physical, cognitive and social anxiety, respectively [56]. In the brain, anxiety during adolescence is related to a reduction in gray matter volume in the limbic regions [57]. More specifically, anxiety sensitivity is associated with the mechanisms of emotional regulation in the connection between the prefrontal cortex and the ventromedial amygdala [58]. In particular, the presence of high anxiety sensitivity in youth is associated with differential functioning of the connectivity between the left lateral subregion of the amygdala and the prefrontal cortex, which is in charge of processing emotions [59]. In general, high levels of anxiety sensitivity and low perceived control of it are associated with more days of alcohol consumption. So those adolescents who are highly vulnerable to anxiety and who think they cannot control it develop maladaptive coping strategies such as drinking [60]. This is partly due to the positive expectations about drinking, such that young people who have the most positive beliefs about the effects of alcohol (for example, believing that drinking reduces tension or improves social situations) and that are more sensitive to the symptoms of anxiety, show a greater increase in drinking alcohol during adolescence [61]. More specifically, social anxiety has been shown to be positively related to drinking problems during puberty [62,63]. Beliefs about the approval of alcohol by the group of close friends promotes its use due to adolescent concern about violating perceived friendship [64]. 

Finally, self-esteem is another important variable for understanding risk behaviors such as drinking, which drive behavior [65] and influence adolescent subjective wellbeing [66]. Self-esteem represents self-acceptance and self-approval of one’s own values and judgments, that is, whether one perceives oneself in favorable or unfavorable terms [67,68]. Several studies have shown how individuals who have high self-esteem feel good about themselves, which leads them to behavior that protects or improves their health and wellbeing [69,70]. On the contrary, low levels of self-esteem have been associated with substance use [70] and the development of an excessive drinking pattern maintained over time [71]. This variable negatively moderates the direct relationship between impulsivity and episodes of excessive drinking by young people. Self-esteem acts as a protective factor in the association between these variables [39], while those with low self-esteem regulate their social behaviors to achieve acceptance of others and avoid rejection [72]. In this regard, adolescents who are insecure in attachments in their relations, and who are marked by low self-esteem [73] and high anxiety, have a higher likelihood of drinking alcohol in an attempt at not being excluded from the group [74]. Other studies have shown that self-esteem has a critical role in adolescent substance use as a mediator in susceptibility to the negative influence of other students [75]. Similarly, a study by Collison, Banbury, Cert, and Lusher [76] showed that both perceived pressure and self-esteem significantly affected attitudes toward drinking, relating low levels of this variable with unrestrictive attitudes toward alcoholic beverages and high levels with restrictive attitudes. This means that the combination of self-esteem and perceived pressure by the peer group can have a decisive role in adolescent attitudes toward alcohol.

Both brain and behavior are considerably pliable in adolescence, so it is an appropriate time to help young people prevent risk behaviors and begin positive health behaviors [7]. Therefore, knowledge of the factors determining adolescent drinking provides the conceptual basis for decision-making and developing preventive strategies directed at reducing the incidence of alcohol abuse, and dissuade the appearance of related problems at the same time [15]. In view of all of the above, the objective of this study was to analyze the variables related to resistance to group pressure in adolescent drinking. 

Impulsivity, self-esteem, anxiety sensitivity and expectations for drinking are individual variables, but at the same time, they are closely linked to the individual’s social development. In line with this proposal, the following study hypotheses were formulated: (H1) There is a positive relationship between perceived pressure to drink alcohol and positive expectations for it by adolescents; (H2) perceived pressure to drink alcohol shows a significant positive association with young people’s anxiety sensitivity to physical, cognitive and social signs; (H3) there is a positive association between perceived pressure for adolescents to drink alcohol and their general impulsivity; and finally, (H4) the pressure that young people perceive to drink alcohol is negatively related to self-esteem.

## 2. Materials and Methods 

### 2.1. Participants

The participants were selected by random cluster sampling. All questionnaires in which random or incongruent answers to control questions inserted for the purpose were discarded from the start. Thus, a total of 1287 students, aged 14 to 18, mean 15.11 (*SD* = 0.91), at several different public high schools in the province of Almeria (Spain) participated in this study. The distribution by sex was 47.1% (*n* = 606) male and 52.9% (*n* = 681) female, with mean ages of 15.12 (*SD* = 0.94) and 15.10 (*SD* = 0.88), respectively. Of these, 55% (*n* = 707) were in third year and 45% (*n* = 577) in fourth year of high school. 51.6% (*n* = 662) of the participants stated that they drank alcohol. The frequency of use was: 30.5% (*n* = 203) said they “had drunk alcoholic beverages very few times in their lives,” 37.8% (*n* = 252) answered “a few times this year”, 25.5% (*n* = 170) stated “a few times this month”, 5.6% (*n* = 37), “a few times a week”, and 0.6% (*n* = 4) said they drank alcohol daily.

### 2.2. Instruments

Sociodemographic data (age, sex, grade, nationality, etc.) were collected on and ad hoc questionnaire. Some questions on their use of alcohol were also included (“Do you drink alcohol?”) and its frequency (“If you answered yes, how often?”) with several answer choices.

The Resistance to Group Pressure in Using Alcohol Questionnaire [77] was used to evaluate resistance to peer group influence. It is comprised of 45 items describing situations in which a young person could be pressured to drink alcohol. Items 1 to 40 are answered on a Likert-type scale from 1 to 4 (from “never” to “always”), and items 41 to 45 are answered on a scale of 1 to 5 to evaluate the subject’s perception of pressure from peers. Three factors were found from clustering the answers to the questionnaire: Resistance to direct group pressure (e.g., “when you are with your friends and you don’t want to drink, if all of them are drinking, you think you have to drink too”) resistance to indirect group pressure (e.g., “when the group does not agree with your decision not to drink, you feel rejected and end up drinking”), and resistance to perceived pressure (e.g., “you are at a meeting and are offered a drink and you don’t want to drink, you say you don’t want to drink and are firm in your decision”). In this study, the reliability indices calculated with the omega, were *ω* = 0.77, *ω* = 0.91 and *ω* = 0.72, respectively. 

For expectations about use, the Alcohol Expectancy Questionnaire-Adolescent Brief [78], Spanish adaptation by Gázquez et al. [79] was employed. This instrument consists of two scales which record adolescent beliefs about the positive and negative social and emotional effects of alcohol: the AEQ-ABp (positive expectations about the effects of alcohol; e.g., “alcohol helps people think more clearly and improves their coordination—you understand things better and do them better”) and AEQ-ABn (negative expectations about the effects of alcohol; e.g., “alcohol worsens people’s reasoning and coordination, you trip, act stupidly, and get a hangover”). They each consist of seven items with answer choices on a five-point Likert-type scale (where 1 is equal to “totally disagree” and 5 “totally agree”). The omega was 0.60.

Anxiety sensitivity was evaluated using the Spanish version for high school students [80] of the Anxiety Sensitivity Index-3 questionnaire [81]. This instrument is made up of 18 items which are answered on a Likert-type scale (from “not at all or hardly” to “very much”, that is, from 0 to 4, respectively). With this questionnaire, three subscales may be found: sensitivity to physical (e.g., “it scares if I feel a pressure in my chest and I can’t breathe well”), cognitive (e.g., “when I can’t keep my mind on my homework, I worry that I might be going crazy”) and social anxiety (e.g., “it is important for me not to give the impression that I’m nervous”). The reliability shown by the scales for this study was *ω* = 0.85 for the cognitive scale, *ω* = 0.78 on the social scale and *ω* = 0.85 for sensitivity to physical anxiety.

The Rosenberg Self-esteem Scale [82] evaluates one’s satisfaction with oneself. It consists of 10 items (e.g., “in general, I feel satisfied with myself”) answered from 1 (“strongly agree”) to 4 (“strongly disagree”) on a Likert-type scale. The reliability shown for the scale in this study was ω = 0.74.

The version adapted for Spanish adolescents by Martínez-Loredo, Fernández-Hermida, Fernández-Artamendi, Carvallo, and García-Rodríguez [83] of the Barratt Impulsiveness Scale BIS-11A; [84] was used for evaluation of general impulsivity. This instrument consists of 30 items that are clustered around two factors: general impulsivity (e.g., “I do things without thinking”) and unplanned impulsivity (p.e.: I concentrate easily). The answer choices follow a Likert-type scale on which the participants must report on the frequency of different behaviors (where 1 is “rarely or never” and 4 is “always or almost always”). Reliability for the unplanned impulsivity factor was *ω* = 0.66 and for the general impulsivity, reliability was *ω* = 0.76.

### 2.3. Procedure

Before data were collected, the directors of the schools were contacted and a meeting was arranged to inform them about the objectives of the research and guarantee the confidential treatment of data. When the sessions had been scheduled, two members of the research team went to the schools to administer the questionnaires. The tests were given in the usual classroom assigned to each group in the presence of their teacher/counselor. At the beginning of the session, before going on to filling in the questionnaires, the students were given the appropriate instructions and were offered time to ask any questions, and the anonymity of their answers was guaranteed, and therefore, that their privacy would be respected in statistical processing. The students filled out the tests anonymously and individually, in an estimated mean time of 25–30 min. In all cases, the ethical standards for research were complied with on an informed consent sheet. The study was approved by the University of Almeria Bioethics Committee (Ref: UALBIO2018/015).

### 2.4. Data Analysis

Pearson’s bivariate correlation was calculated to identify the relationships between variables. From this first approximation, data were extracted for an in-depth analysis of the relationships by Bayesian correlation pair analysis, estimating the Bayes Factor (BF_10_) to compare the alternative hypothesis to the null hypothesis in each pair of variables. JASP software (Version 0.11.1, Amsterdam, Netherlands) was used for this [85]. In addition, simple mediation analyses were performed, taking as dependent variables those identified as risk variables (with strong positive association after the Bayesian hypothesis comparison) for group pressure to drink alcohol. In each case, self-esteem was entered as a possible mediator (with a negative association with group pressure). The PROCESS macro for SPSS (version 2.16.3, Ohio, United States) [86] was used to process the mediation models with bootstrapping with coefficients estimated from 5000 bootstraps. To examine the reliability of the instruments used for data collection, McDonald’s Omega coefficient [87] was estimated, following the proposal and instructions of Ventura-León and Caycho [88].

## 3. Results

Table 1 shows the bivariate correlation matrix with the Pearson’s correlation coefficient. It may be observed that perceived group pressure was positively related to positive expectations about drinking, with the three anxiety sensitivity factors (cognitive, social and physical), and with general impulsivity. On the contrary, perceived group pressure correlated negatively with self-esteem. Similarly, self-esteem was correlated negatively to the rest of the variables, except negative expectations about drinking, with which it was unrelated. Furthermore, the positive expectations, anxiety sensitivity and impulsivity factors were correlated positively to each other.

**Figure 1:**
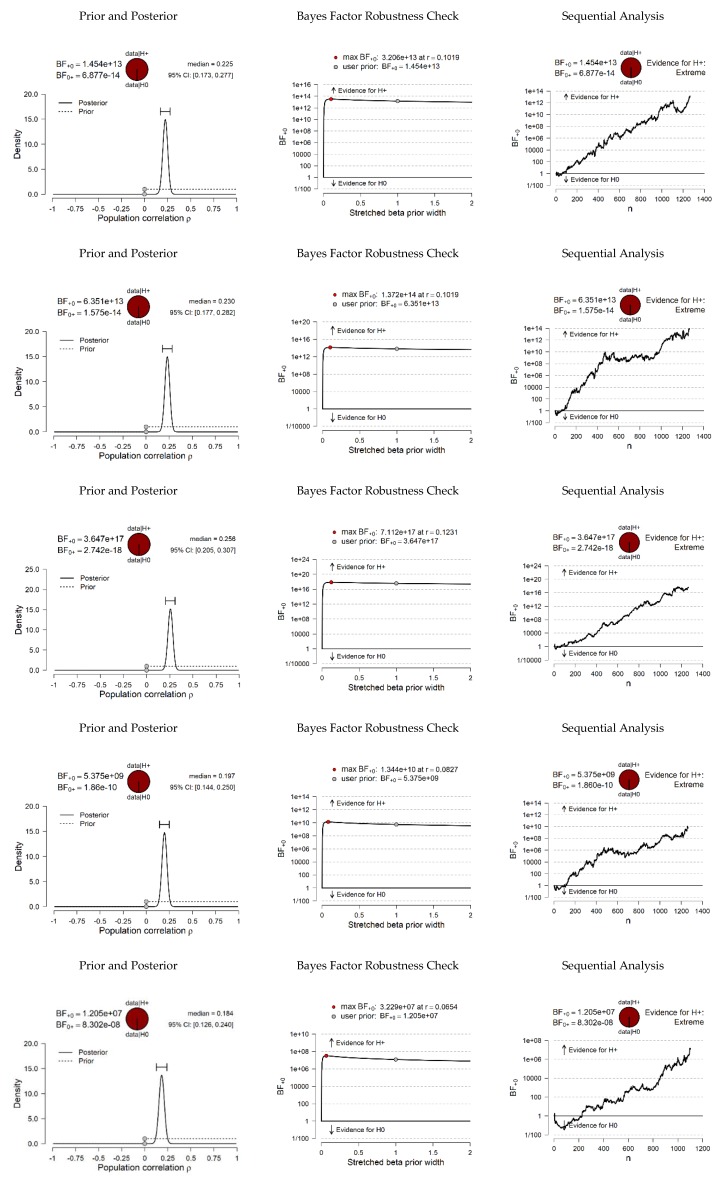
Bayesian correlation pairs. (Note: for all tests, H_1_ specifies that the correlation is positive).

**Figure 2:**
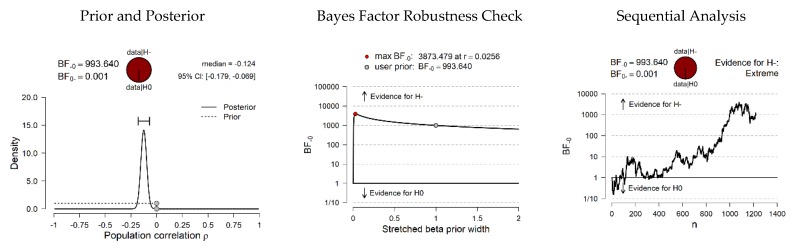
Bayesian correlation pairs. (Note: H_1_ specifies that the correlation is negative).

Based on the relationships identified, and supported by the literature on the subject, a series of hypotheses were posed to be tested by Bayesian correlation pair analysis (Figure 1 and Figure 2). Specifically, it was evaluated whether it is more probable that data occur under the null hypothesis (which argues that there is no linear association between variables), or under the alternative hypothesis (which argues that there is a positive or negative association, depending on the pair of variables in each case). 

First, a BF_10_ was found for the PP ^[+]^ PE (positive expectations ↔ perceived pressure) pair, which showed that the data were 1.454 × 10^13^ times more probable under H_1_ than H_0_, providing extreme evidence in favor of a true correlation other than zero, and that the true correlation was between 0.173 and 0.277 at a 95% confidence interval. Moreover, in the comparison of the PP ^[+]^ ANXc (anxiety cognitive factor ↔ perceived pressure) pair, the BF_10_ suggested that data were 6.351 × 10^13^ times more likely under H_1_ than under H_0_, providing extreme evidence in favor of a true correlation other than zero, and that the true correlation was between 0.177 and 0.282 at a 95% confidence interval. For the PP ^[+]^ ANXs (anxiety social factor ↔ perceived pressure), the BF_10_ found showed that data were 3.647 × 10^17^ times more probable under H_1_ than H_0_, providing extreme evidence in favor of H_1_ and that the true correlation was between 0.205 and 0.307 at a 95% CI. For the PP ^[+]^ ANXp (anxiety physical factor ↔ perceived pressure) pair, the BF_10_, showed that the data were 5.375 × 10^9^ times more likely under H_1_ than under H_0_, providing extreme evidence in favor of H1 at a 95% CI (0.144, 0.250). For the PP ^[+]^ GI (general impulsivity ↔ perceived pressure), the BF_10_ showed that the data are 1.205 × 10^7^ times more likely under H_1_ than under H_0_, providing extreme evidence in favor of H_1_ at a 95% CI (0.126, 0.240).

Finally, as shown in Figure 2, the hypothesis comparison for PP ^[−]^ SE (self-esteem ↔ perceived pressure), resulted in a BF_10_ showing that the data are 993 times more likely under H_1_ than under H_0_, also providing extreme evidence in favor of H_1_ at a 95% CI (−0.179, −0.069). 

### Mediation Models of Self-Esteem on the Relationship between the Risk Variables and Perceived Group Pressure for Adolescent Drinking

**Figure 3:**
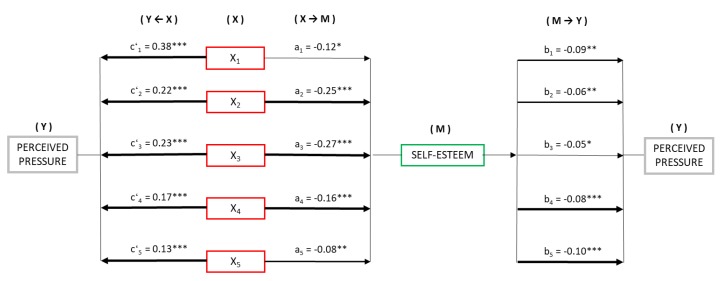
Mediation model of self-esteem on the relationship between risk variables and perceived group pressure to drink alcohol (Note: X_1_ = PE, X_2_ = ANXc, X_3_ =ANXs, X_4_ =ANXp, X_5_ = GI. * *p* < 0.05, ** *p* < 0.01, *** *p* < 0.001).

Based on these results, we saw the need to find out whether self-esteem could be mediating in the relationship between the risk variables and perceived group pressure on adolescents to drink alcohol. Therefore, simple mediation models were computed. Figure 3 presents the mediation models, taking as predictors in each case, the risk variables: positive expectations for drinking alcohol (X_1_), cognitive factor (X_2_), social factor (X_3_), and physical factor (X_4_) of anxiety sensitivity and general impulsivity (X_5_). Self-esteem was the mediator (M) as a factor common to all the models and perceived pressure as the dependent variable (Y).

In the first place, significant negative relationships were observed between the predictor variables and self-esteem (M): PE (B = −0.12, 95% CI (−0.232, −0.010)), ANXc (B = −0.25, 95% CI (−0.325, −0.193)), ANXs (B = −0.27, 95% CI (−0.335, −0.210)), ANXp (B = −0.16, 95% CI (−0.219,−0.102)), and GI (B = −0.08, 95% CI (−0.138, −0.033)). 

Similarly, estimation of the direct effects X→Y, revealed significant relationships with the risk variables: PE (B = 0.38, 95% CI (0.289, 0.478)), ANXc (B = 0.22, 95% CI (0.162, 0.279)), ANXs (B = 0.23, 95% CI (0.180, 0.292)), ANXp (B = 0.17, 95% CI (0.120, 0.221)), and GI (B = 0.13, 95% CI (0.087, 0.176)), on perceived pressure (Y). In addition, the estimation of M→Y showed a significant negative relationship in all cases for self-esteem on perceived pressure (Y), varying from B = 0.05 to 0.10.

Finally, the analysis of indirect effects (X→M→Y) with bootstrapping found significant values in all five models computed: PE (B = 0.01, SE = 0.006, 95% CI (0.001, 0.027)), ANXc (B = 0.01, SE = 0.007, 95% CI (0.005, 0.034)), ANXs (B = 0.01, SE = 0.007, 95% CI (0.003, 0.031)), ANXp (B = 0.01, SE = 0.004, 95% CI (0.005, 0.025)), and GI (B = 0.008, SE = 0.003, 95% CI (0.003, 0.017)).

## 4. Discussion 

Adolescent drinking is a widely extended reality [1], which is harmful to health [4,5,6,7,8,9] and to teenage psychosocial development [10,11,12,14]. This study analyzed the role of self-esteem, impulsivity, anxiety sensitivity and expectations about drinking on perceived pressure on adolescents to drink alcohol. The first hypothesis (H1) was posed based on the literature which has shown that favorable beliefs about drinking alcohol diminish adolescents’ ability to resist, especially when they think it is going to have gratifying social consequences [28]. In the light of the results, this was confirmed, which means that young people who perceive multiple benefits of alcohol, feel the urge to drink it. Thus, the increase in the need for affiliation with peers [19], along with positive expectations of greater importance during adolescence, related to decreased social inhibition and improvement in socio-affective skills [24,47] lead to increased pressure to drink alcohol. 

Our second hypothesis was also confirmed (H2). As already demonstrated in other studies [60,61,62], anxiety sensitivity is related to teenage drinking. This could be because they become more easily excited by altered physiological, cognitive or social signs and try to reduce the tension in a maladaptive way [61], partly allowing themselves to be guided by the prescription of their peers, who are a very strong influence [20,22] especially when the adolescent fears violating perceived friendship [58].

The impulsivity variable has been shown to be related to more drinking during adolescence [30,38,39,40,41,42], as the reduction of the capacity for self-regulation in this stage increases their vulnerability to the influence of others in substance use [46]. Finally, H3 was proposed, which resulted in a higher probability of the existence of this relationship, than of its absence. This means that young people with greater difficulty in controlling their impulsive tendencies are more receptive to pressure to drink alcohol. 

The last hypothesis (H4) posed referred to the negative association between self-esteem and perceived pressure. Self-esteem is one’s acceptance of one’s own values, satisfaction with oneself [67]. This makes young people with higher self-esteem show sufficient confidence in themselves and their ideals to not depend on social acceptance [72] and are able to make their own decisions about whether to drink. The results confirmed this hypothesis, which implies that young people with low levels of self-esteem, in an attempt not to feel rejected by the group, are more susceptible to group pressure to drink alcohol. 

The mediating role of self-esteem in the direct positive association of the risk variables with perceived pressure was analyzed based on the various signs of association found between the variables considered risks (that is, anxiety sensitivity, impulsivity and positive expectations) and self-esteem with the perception of pressure to drink alcohol. The results showed that self-esteem mediates the relationship between physical, cognitive and social anxiety sensitivity, impulsivity and positive expectations with perceived pressure in adolescence. Studies such as the one by Collison et al. [76], had already shown the role of self-esteem in young people’s attitudes toward drinking, in spite of the pressure for them to drink. Likewise, Yan et al. [75] identified the mediating role of self-esteem on susceptibility to influence of peers. The results of the mediation model confirm these findings, demonstrating that the most sensitivity to social demands for drinking alcohol made on young people with high scores in impulsivity, susceptibility to anxiety and who think that drinking benefits them, are attenuated when the adolescent has high self-esteem. 

It is worth mentioning some limitations. In the first place, the dependent variable in this study was perceived pressure to drink alcohol and not drinking itself. Therefore, caution should be taken in interpreting the findings. The feeling of being under more pressure to drink does not necessarily imply drinking. Although it is true that perceived pressure by the peer group is a variable with great weight in this stage, where young people show a strong need for affiliation with peers, other variables not included in this study could be equally related to the real use rate. Therefore, future studies should also include variables such as parenting styles, self-efficacy for resisting drinking and perceived social support. Furthermore, in spite of the large number of studies relating the variables dealt with in this article, there are no previous studies analyzing them all at the same time, which diminishes the possibility of comparing our results. Another limitation is that the sample was taken from high schools, so it did not reach adolescents with school absenteeism or who did not continue their studies beyond primary school. Future studies are therefore recommended to use sampling techniques that make it possible to gain access to this sector of the population. Finally, we should mention the research method, which was cross-sectional and exploratory and did not allow us to establish causal relationships. Therefore, future studies should include longitudinal evaluation of the youths, which would enable changes in perceived pressure to drink alcohol and the effects generated by the rest of the variables analyzed to be found at different times during this stage of their development. 

## 5. Conclusions

Drinking alcohol in adolescence is a public health problem. In view of the pliability of behavior during this stage, prevention is possible and action can be taken on such detrimental behavior. Therefore, knowledge of the variables associated with its development and prevention are fundamental in approaching drinking by minors. The results of this study show how individual variables such as anxiety sensitivity, impulsivity and positive expectations act as risk variables for perceived pressure to drink alcohol, while self-esteem mediates these relationships, reducing their effect. 

Given the strong need for affiliation of young people, it is hard to control grouping and influence of the peer group on adolescent behavior, such as drinking alcohol. However, it is possible to act on individual variables such as those analyzed here, enabling their capacity to resist urging from classmates. Therefore, the practical application of the results is related to the possibilities the data found provide for designing prevention and intervention programs in adolescent populations directed at reducing risk behaviors such as drinking. 

## Figures and Tables

**Table 1:** Bivariate correlations.

		PE		EN		SE		ANXc		ANXs		ANXp		GI	
PE	Pearson’s r	—													
	Upper 95% CI	—													
	Lower 95% CI	—													
EN	Pearson’s r	0.251	***	—											
	Upper 95% CI	0.302		—											
	Lower 95% CI	0.200		—											
SE	Pearson’s r	−0.059	*	−0.032		—									
	Upper 95% CI	−0.003		0.024		—									
	Lower 95% CI	−0.114		−0.087		—									
ANXc	Pearson’s r	0.133	***	−0.076	**	−0.218	***	—							
	Upper 95% CI	0.186		−0.021		−0.164		—							
	Lower 95% CI	0.079		−0.130		−0.270		—							
ANXs	Pearson’s r	0.123	***	0.042		−0.242	***	0.606	***	—					
	Upper 95% CI	0.177		0.096		−0.189		0.640		—					
	Lower 95% CI	0.069		−0.013		−0.294		0.570		—					
ANXp	Pearson’s r	0.101	***	−0.020		−0.157	***	0.671	***	0.515	***	—			
	Upper 95% CI	0.155		0.035		−0.102		0.700		0.554		—			
	Lower 95% CI	0.047		−0.074		−0.211		0.640		0.473		—			
GI	Pearson’s r	0.214	***	0.060	*	−0.096	**	0.312	***	0.258	***	0.209	***	—	
	Upper 95% CI	0.269		0.118		−0.037		0.364		0.312		0.264		—	
	Lower 95% CI	0.157		0.001		−0.155		0.258		0.202		0.152		—	
PP	Pearson’s r	0.226	***	0.022		−0.125	***	0.231	***	0.257	***	0.197	***	0.184	***
	Upper 95% CI	0.277		0.077		−0.069		0.282		0.308		0.250		0.240	
	Lower 95% CI	0.173		−0.033		−0.180		0.178		0.205		0.144		0.126	

Note. PE = Positive expectations, EN = Negative expectations, SE = Self-esteem, ANXc = Anxiety cognitive factor, ANXs = Anxiety social factor, ANXp = Anxiety physical factor, GI = General impulsiveness, PP = Perceived pressure.* *p* < 0.05, ** *p* < 0.01, *** *p* < 0.001.
